# A Study on the Impacts of Slot Types and Training Data on Joint Natural Language Understanding in a Spanish Medication Management Assistant Scenario

**DOI:** 10.3390/s22062364

**Published:** 2022-03-18

**Authors:** Surya Roca, Sophie Rosset, José García, Álvaro Alesanco

**Affiliations:** 1 Aragón Institute of Engineering Research (I3A), University of Zaragoza, 50018 Zaragoza, Spain; jogarmo@unizar.es (J.G.); alesanco@unizar.es (Á.A.); 2 Laboratoire Interdisciplinaire des Sciences du Numérique, CNRS, Université Paris-Saclay, 91405 Orsay, France; sophie.rosset@lisn.upsaclay.fr

**Keywords:** intent detection, slot filling, medication management scenario, joint natural language understanding, training data

## Abstract

This study evaluates the impacts of slot tagging and training data length on joint natural language understanding (NLU) models for medication management scenarios using chatbots in Spanish. In this study, we define the intents (purposes of the sentences) for medication management scenarios and two types of slot tags. For training the model, we generated four datasets, combining long/short sentences with long/short slots, while for testing, we collect the data from real interactions of users with a chatbot. For the comparative analysis, we chose six joint NLU models (SlotRefine, stack-propagation framework, SF-ID network, capsule-NLU, slot-gated modeling, and a joint SLU-LM model) from the literature. The results show that the best performance (with a sentence-level semantic accuracy of 68.6%, an F${}_{1}$-score of 76.4% for slot filling, and an accuracy of 79.3% for intent detection) is achieved using short sentences and short slots. Our results suggest that joint NLU models trained with short slots yield better results than those trained with long slots for the slot filling task. The results also indicate that short slots could be a better choice for the dialog system because of their simplicity. Importantly, the work demonstrates that the performance of the joint NLU models can be improved by selecting the correct slot configuration according to the usage scenario.

## 1. Introduction

Natural language understanding (NLU) is a critical component in the fluent and successful conversations between chatbots and their users. The interest in NLU has recently increased, as proven by the increasing number of conversational systems in numerous fields that attempt to improve our quality of life [1,2]. Inside a specific domain, the NLU involves the tasks of slot filling and intent detection. Slot filling attempts to tag the words in a sentence with the slot types [3] and intent detection can be defined as the action of labeling the sentence based on predefined intents. An example of intent detection and slot filling in an NLU model that receives, as an input, the sentence “I would like to add paracetamol” is to obtain the intent ADD and extract the key-value slot {medication = paracetamol}.

In previous years, several studies were carried out to improve and evaluate the performance of NLU tasks. Former works evaluated the impact of using different techniques to improve slot filling [4,5,6] and intent detection [7,8]. Interestingly, research in joint NLU (jointly learning both intent detection and slot filling) achieved better results in both tasks [9,10]. Moreover, Wang et al. [11] proposed a new joint model to improve the slot tagging task by combining sequence labeling with a deep bidirectional transformer. Word embeddings were also studied for the improvements of the NLU task in the biomedical domain [12,13,14]; researchers observed that embeddings trained with specific domains do not have better performances in comparison with the general domain. Sushil et al. [15] studied the use of further language model pretraining, lexical match algorithms, supplementing lexical retrieval, and trained retriever modules in the clinical domain; they observed that the proposed extensions did not show significant improvement and that methods should be developed to augment fundamental domain knowledge from textual corpora.

Recent works focused on the importance of selecting the data to train the models in a low-data regime, with researchers observing that the selection criteria can have a strong influence on the performance of the model [16]. The data used in low-data resource scenarios, such as the biomedical domain, are generated in order to train the models, which could have different characteristics. Furthermore, the decision of slot tagging is not always direct. In fact, there are multiple ways to tag the information in a specific context. To address this gap, this article presents a detailed analysis of the impact of the sentence length of the dataset and the decision in the slot tags, and how they can influence the performance of the NLU models. To the best of the authors’ knowledge, a detailed study evaluating the impact of slot tagging and dataset length on intent detection and slot filling for the medication domain in Spanish has not been carried out before.

This study focuses on what is best for joint NLU models in a medication scenario using the Spanish language, the analysis being two-fold: (1) the impact of having complex but shorter concepts compared to having simple and fewer but longer concepts; (2) the impact of generating datasets with long and short sentences.

## 2. Materials and Methods

### 2.1. Study Overview

Given a sentence consisting of a collection of *n* words defined as *w* = {*w${}_{1}$*, *w${}_{2}$*,…, *w${}_{n}$*}, the main purpose of our study is to predict the correct intent *i*, where *i*∈ {ADD, SEE,…, BYE} and the set of slots *s* = {*s${}_{1}$*, *s${}_{2}$*,…, *s${}_{n}$*} associated with each word *w${}_{k}$*, where *s${}_{k}$*∈ {O, B-medication, I-medication,…, B-posology, I-posology} if we are in the long-slot configuration or *s${}_{k}$*∈ {O, B-medication, I-medication,…, B-timeExpressions, I-timeExpressions} if we are in the short-slot configuration. Slots use the IOB (inside, outside, beginning) format.

**Figure 1:**
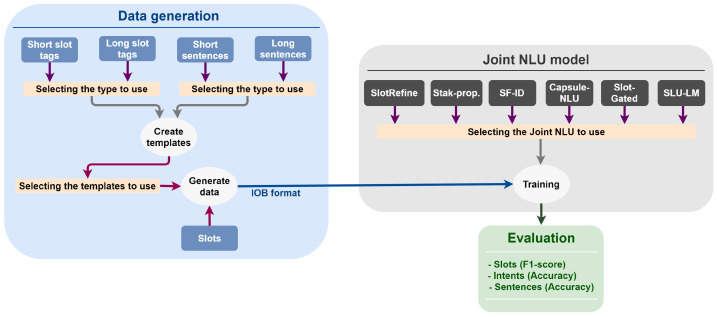
Overview of the system.

The system used in this study (shown in Figure 1) consists of two components: data generation and the joint NLU model. To determine the best configuration of data characteristics, we generated different datasets in the medication domain and tested them with different joint NLU models. Generally, it is quite challenging to gather a big corpus in the medical domain due to privacy issues (there is no access to corpus obtained from conversations with real patients in real scenarios). For this reason, we resorted to generating datasets that were close to the ones in real life.

The details of the two components are described in the following subsections. Additionally, we present in detail the intents and slots proposed for this scenario and the real test data obtained from a group of real users.

### 2.2. Data Generation

We generated a total of four datasets in Spanish to estimate the influence of slot tagging and sentence length. The four datasets are: two datasets with long sentences, one tagged with long slots and the other tagged with short slots, and two datasets with short sentences, one tagged with long slots and the other tagged with short slots. Table 1 summarizes the characteristics of the different datasets generated. These datasets were used as the input of the joint NLU models. All sentences are tagged using the IOB format.

We wanted to compare two different slot types: (1) short but complex; (2) long but simple and fewer slots. Therefore, we used templates created for each dataset with the corresponding slot tagged in each template. Then, we defined one file for each slot, and we used the method proposed by Boulanger [17] to generate the training and the development dataset for each combination. The method is based on a simple filling pattern for obtaining all of the possible sentence combinations and then sampling the generated sentences through a series of Euclidean divisions. The number of templates used to generate each dataset is shown in Table 1. An example of a long sentence is “I want to add $medication $duration”, where a word after $ character means it is a slot, and an example of a short sentence is “Add $medication”. After using the pattern filling, an example of a long sentence generated is “I want to add ibuprofen for 3 weeks” and an example of a short sentence is “Add ibuprofen”.

We also wanted to compare two different sentence lengths. Hill et al. [18] compared the differences between human–human and human–chatbot interactions in the general domain, showing that a human–human conversation has a sentence length average of 7.95 words, whereas a human–chatbot conversation has a sentence length average of 4.29. We used these length averages as references, creating datasets with short sentences a bit shorter (4.1) and long sentences longer (between 9.1 and 10.9), with an average length difference of 5.9 between short and long sentences. Training datasets have, in total, 24,270 sentences, while development datasets have 5915 sentences.

Examples of the long sentences include the following (examples are translated from Spanish for ease of reading):

- The doctor told me to take risedronato;
- Four puffs before going to bed for twenty-five days;
- The nurse told me to take rilast;
- Until 24 February 2023, two drops every two weeks.

Short sentence examples include the following:

- Delete medication;
- Summary of 8 days;
- Add doxidina;
- Yes, I took it.

### 2.3. Test Data

For the test data, we collected real data from a total of 14 users through the interactions with a chatbot. The chatbot had basic knowledge to ask questions related to medication management and continue the conversation with the user. The total number of sentences in the test data was 382, with a sentence length average of 2.70, and the total number of words was 1031. As for the vocabulary of the test data—there were 11 unknown words compared to the Spanish Wikipedia corpus. The unknown words were due to typographical errors, i.e., holaa (hi), ningunooo (none), pliss (please), and medication names (some with typographical errors): pharmagrip, eutirox, noctamid, norotil, traumel, urorec, uroret, espidifen.

Some examples from test data are the following (translated from Spanish):

- See my medications;
- Well I want to add paracetamol;
- Delete frenadol;
- Can you show me my medication?

### 2.4. Intent Detection

We defined intent related to the medication scenario and additional intent related to the comprehension of the user’s answers in a chatbot conversation. As our scenario focused on the task of medication management, we defined the intent as shown in Table 2.

### 2.5. Slot Filling

**Figure 2:**
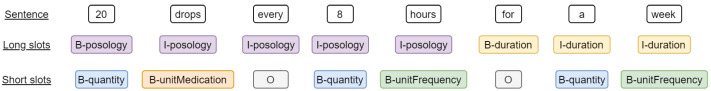
Example of slot filling with long and short slot configurations (using the IOB format).

One of the purposes of this study was to determine the best slot tagging strategy, either having complex but shorter concepts or having simple and fewer but longer concepts. For our medication management scenario, we defined two slot configurations (shown in Table 3). Long slots were designed to cover posology (i.e., the branch of pharmacology that determines the appropriate doses of drugs and medicines) information in one slot, whereas short slots split the posology into different concepts, such as quantity, unit medication, unit frequency, and time expressions. The duration of the treatment was also defined as one slot in a long slot configuration, whereas a short slot configuration split the duration in quantity and unit frequency. The medication and date slots were the same for both configurations. The date slot refers to a specific day (for example, to indicate when the treatment starts). An example of these two slot configurations is shown in Figure 2, where posology (“20 drops every 8 h”) can be expressed using quantity (“20” and “8”), unit medication (“drops”), and unit frequency (“hours”); duration (“for a week”) can be expressed using quantity (“a”) and unit frequency (“week”). As mentioned previously, the sentences are tagged using the IOB format.

### 2.6. Joint NLU Model

In this study, we used joint NLU models proposed in the literature to evaluate the effects of the generated datasets in intent detection and slot filling. The maximum number of epochs (one pass of the entire training dataset through the algorithm) was set to 100 for all simulations in this study. The rest of the hyperparameters not specified are with the default value. We used the following joint NLU models with their respective configurations.

- SlotRefine [19]: is a two-pass refine mechanism that uses the B-tags of the first pass as input for the second pass. The results were obtained with a batch size of 32 and 64, a learning rate of 0.0025 and 0.001, a hidden size of 80 and 96, a filter size of 80 and 96, and a number of heads of 8 and 16. Furthermore, the attention dropout was set to 0.05, patience set to 0, remove numbers was set to true, and the multiply embedding mode was set to “none”.
- Stack-propagation framework [20]: is a joint learning scheme, which first uses a self-attentive encoder, performs a token-level intent detection, and finally leverages the token-level intent information to improve the slot filling decoder. The results were obtained with a word embedding dimension of 32 and 256.
- SF-ID network [21]: has a model architecture that is based on bi-directional LSTM (BLSTM) and uses two subnets: SF subnet and ID subnet. SF-ID network also includes a CRF layer to jointly decode the labels of the utterance. We obtained the results with four configurations: SF-first with and without CRF, and ID-first with and without CRF. The early stop was disabled.
- Capsule-NLU [22]: combines dynamic routing and re-routing processes with three different types of capsules (WordCaps, SlotCaps, and IntentCaps) to accomplish intent detection and slot filling. We obtained the results with two configurations: using dynamic routing and re-routing processes and using only dynamic routing processes. The early stop was disabled.
- Slot-gated modeling [9]: is an attention-based recurrent neural network (RNN) model. We obtained the results with the two different models proposed in the slot gate mechanism: slot attention, and intent attention approach, and only intent attention approach. The early stop was disabled.
- Joint SLU-LM model [23]: is a joint online spoken language understanding (SLU) and language modeling (LM) with RNN. We obtained the results with two different configurations: recurrent intent context and both local and recurrent intent context. The maximum sequence length was set to 50, DNN at the output layer was set to true, steps per checkpoint were set to 1017, and the maximum training steps were set to 101,700.

### 2.7. Evaluation

The results were evaluated using the micro F${}_{1}$-score with a chunk (a group of words grouped with the same tag that has discrete grammatical meanings)-level in the slot filling and the accuracy score for intent detection and sentence-level semantic frame. The sentence-level semantic frame indicates the general performance of the model, taking into consideration both tasks: intent detection and slot filling need to be correct in a sentence to consider the sentence correctly predicted. The F${}_{1}$-score (defined by the Equation (1)) considers the true positives (TP, the model predicts the chunk correctly), the false positives (FP, the model predicts a chunk where "there is not"), and the false negatives (FN, the model predicts there is not a chunk where there is one). The best value of the F${}_{1}$-score is 1, and the worst is 0. Accuracy is the ratio of the correct predictions to the total predictions made (Equation (2)). For accuracy, 1 is the best result, and 0 is the worst.

Furthermore, the Wilcoxon signed-rank test (a non-parametric statistical hypothesis test that compares two paired groups to assess whether the sets of pairs are significantly different from each other) was used to evaluate the statistical significance of the outcomes for continuous variables. Null hypothesis: there was no significant difference in F${}_{1}$-score/accuracy results between short and long slots/sentences. If the *p*-value is lower than 0.05, the null hypothesis is rejected, and the results are considered significantly different. All statistical analyses were conducted using R software, version 4.0.3 [24].

(1) $$F_{1}=2\cdot \frac{precision\cdot recall}{precision+recall}=\frac{2\cdot TP}{2\cdot TP+FP+FN}$$

(2) $$Accuracy=\frac{TP+TN}{TP+TN+FP+FN}$$

The error of each slot was calculated using Equation (3). Error-values of 0 or close to 0 mean that the model was able to predict the presence and the absence of the slot in the sentences, and values of 1 or close to 1 show a model that is not able to predict the slot correctly.

(3) $$Error=\frac{FP+FN}{TP+TN+FP+FN}$$

## 3. Results

The results for slot filling, intent detection, and sentence-level semantic frame are summarized in Table 4, Table 5 and Table 6, where the best results for each model are highlighted in bold. The SlotRefine results shown in the tables were the best results obtained for each dataset from all possible configurations. Stack-propagation results used a word-embedding dimension of 32. The best results for SF-ID were obtained with ID-first and without the CRF layer. Capsule-NLU results used dynamic routing and re-routing processes. The best results for the slot-gated modeling were obtained with the intent attention approach. Joint SLU-LM results used both local and recurrent intent contexts. Overall, the best results were obtained using short sentences and short slots for most joint NLU models.

We performed statistical testing for slot tags and sentence lengths. If the *p*-value is lower than 0.05, we can conclude that the results are significantly different.

Slot tags:

- Results for long sentences and long slots versus long sentences and short slots (*p* = 0.28) are not significantly different.
- Results for short sentences and long slots versus short sentences and short slots (*p* = 0.004) are significantly different.

Sentence length:

- Results for long sentences and long slots versus short sentences and long slots (*p* = 0.12) are not significantly different.
- Results for long sentences and short slots versus short sentences and short slots (*p* = 0.04) are significantly different.

When we compare long to short sentences, and short to long slots, the best results for slot filling and sentence accuracy are achieved with short sentences and short slots when SlotRefine is used (76.4 and 68.6%, respectively), and the best result for intent detection are achieved with long sentences and long slots when SlotRefine is also used (with an accuracy of 81.4%).

### 3.1. Datasets

**Figure 3:**
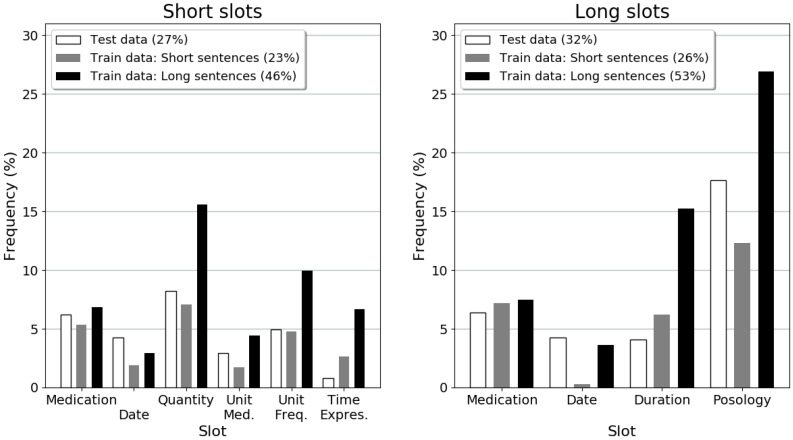
Composition of the datasets (short slots datasets (**left**) and long slots datasets (**right**)) in terms of slot tags and tagged slot proportion (the legend percentage).

In order to observe the differences and similarities in the datasets, Figure 3 shows the frequency of the slots in the test and generated datasets. The percentage of the number of slots in the datasets is also included. We observed that short sentences have a percentage of the number of slots in the dataset similar to the test data, compared to long sentences.

### 3.2. Errors

The percentage of errors for slot filling can be observed in Table 7 and Table 8. Observing the slots that are in both configurations, the medication slot shows the highest value of errors; short sentences with short slots had the best configuration for the prediction of this slot for 4 out of 6 models. The date slot had the highest errors when using short slots and short sentences.

We can observe that, comparing duration and posology with quantity, unit medication, unit frequency, and time expressions, long slots have an average error of 9.96% compared to 6.18% for short slots. We observe that, for long slots, short sentences have an average error of 40.60%, and long sentences have 39.85%. Furthermore, for short slots, short sentences have an average error of 32.60%, and long sentences have 27.13%. Based on these results, we can conclude that joint NLU models trained with long slots yield worse results than the models trained with short slots.

### 3.3. Model Performance

**Figure 4:**
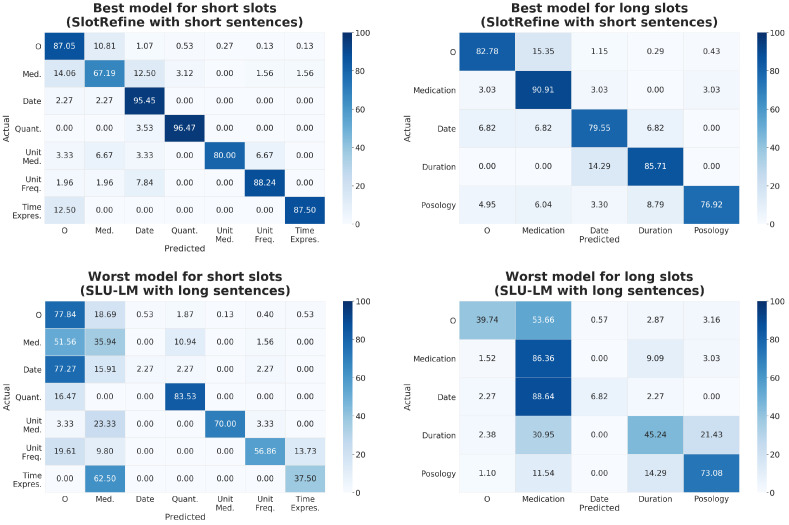
Confusion matrices for the best and the worst models for short and long slots (in %).

**Figure 5:**
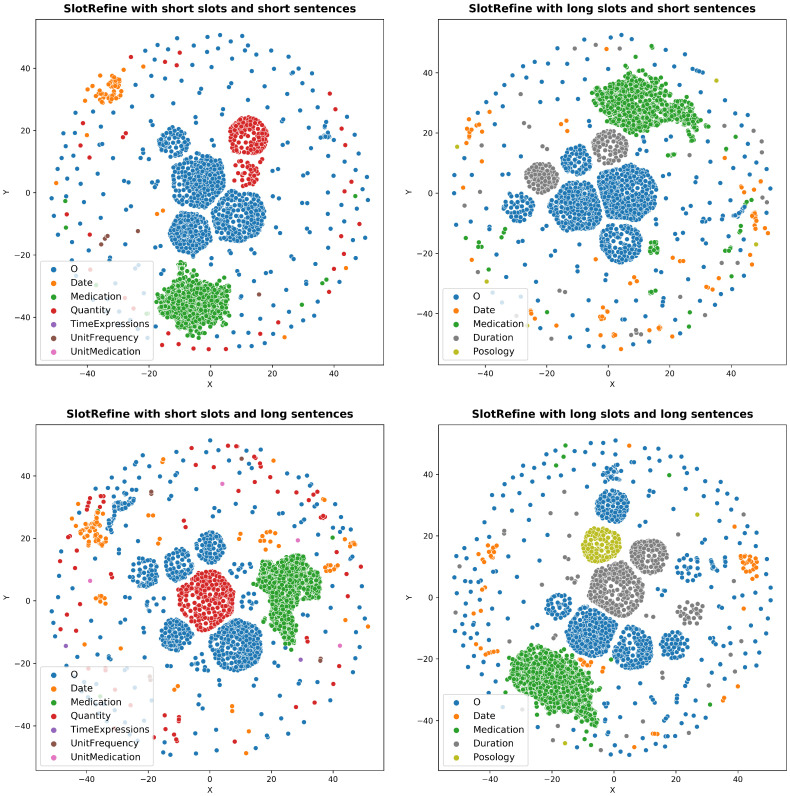
t-SNE representations for the best models for short/long slots and shot/long sentences.

The performances of the models were analyzed using the confusion matrix and the t-SNE representation. The confusion matrices for the best and the worst models are shown in Figure 4. We observe that, for the best model trained with short slots, medication and unit medication were the slots that had more errors, whereas date and medication were the slots that had more errors for the worst model. In addition, the best model trained with long slots showed more errors in posology slots, and the worst model trained with long slots had more errors in date slots. Observing the best model predictions for short slots, nada (nothing) was wrongly predicted ten times, ninguno (none) six times, medicamentos (medications) five times, and Hola (Hi) five times. All of them were tagged as medication and should have been tagged as “O”. It seems that the model interpreted frequent words, such as “nothing” or “Hi”, as medication. This fact may increase the number of mistakes for this slot type.

t-SNE representations are shown in Figure 5. We used the vocabulary from training, development, and test data to represent their transforms with the different models. We compared the best model for each combination of slots and sentences. With this visual evaluation, we can observe how scattered the vocabulary is in each model. The models trained with short sentences show the tags as more compact and clustered, which may be the reason why these models obtained the best results.

## 4. Discussion

In this study, we evaluated six joint NLU models trained with four different datasets. In all of the models, short slots (with an average of 54.7) showed better results in the case of slot filling compared to long slots (average of 33.7). This result may be due to the fact that short slots are more specific and avoid ambiguities that may appear in long slots. Sentence length did not seem to affect slot filling results, with an average of 44.2 for both short and long sentences. It seems that slot tags and sentence lengths were slightly significant variables for intent detection since short slots have an average of 56.8, long slots 58.3, short sentences 58.6, and long sentences 56.5. In the case of sentence-level, the outcomes showed that short sentences (with an average of 36.6) had slightly better performance than long sentences (average of 34.5), and 5 out of 6 models yielded better results with short sentences. The slot tags did not affect the sentence level, with an average of 35.8 for short slots and 35.4 for long slots. To summarize, the slot type seems to be a variable that affects the slot filling outcome but not intent detection or sentence-level outcome. Sentence length seems to be a variable that slightly affects sentence-level and intent detection outcomes, but not the slot filling outcome.

As observed in Figure 3, short sentences and test data have a similar frequency of slots in the sentences, whereas long sentences have a significantly high frequency. Long sentences have more combinations of slots in the same sentence, which may increase the complexity and show a slightly negative impact on the performance of the models. We noted that, for the slots, such as quantity, unit medication, unit frequency, and time expressions, the percentage of error was more or less the same while using different joint NLU models (as observed in Table 7). This can be explained by the fact that short slots may decrease the complexity of the task, observing a better performance in the joint NLU models, whereas long slots, such as duration or posology, increase the difficulty, with different error percentages between models.

### Impact on Dialogue System

Another fact to consider is that the NLU model is part of dialog management. After obtaining the relevant information in the sentence by the NLU model, the slots tagged need to be processed to save the information accordingly. In this case, if, for example, the posology is tagged with long slots, it needs complex post-processing with regular expressions where the different values of the posology need to be obtained. Nevertheless, short slots can simplify the post-processing task because they are already divided into small pieces of information, being a better choice for the complete task, the dialog system.

## 5. Conclusions

This work analyzed the impact of slot tagging and training sentence length using four different datasets generated specifically for this study. The findings contributed toward understanding the effects of slot tagging and the characteristics of the generated data in joint NLU models designed to be used in chatbots, specifically for medication management scenarios in Spanish. A total of six joint NLU models were used to analyze the influence of the training data. In our analysis, short slots significantly yielded better results for slot filling outcomes. It seems that training sentence length slightly affects the performance of the models at intent detection and the sentence level. Our analysis obtained the best outcomes with short slots and short sentences using SlotRefine as the NLU model. In future work, we will investigate the impacts in other languages and domains.

## Figures and Tables

**Table 1:** Datasets summary.

Dataset	Number of Templates	Training Sentence Length (Average)	Training Total Words	Development Sentence Length (Average)	Development Total Words
Long sentences and short slots	13,942	10.79	261,946	10.92	64,599
Long sentences and long slots	1756	9.06	219,949	9.23	54,590
Short sentences and short slots	367	4.06	98,574	4.11	24,337
Short sentences and long slots	367	4.10	99,428	4.13	24,403

**Table 2:** Intent defined for the medication scenario.

Intent	Category	Example
ADD	Medication management	I want to add medication
SEE	Medication management	I want to see my medication
DEL	Medication management	I want to delete a medication
PRO	Medication management	I want to see my summary of medication intakes
INT	Medication management	I have taken the medication of today
OPT	Help	What can I do?
YES	Answer	Yes
NO	Answer	No
NEUTRAL	Answer	Whatever you want
BCK	Answer	Cancel
HELLO	Greetings	Hi
BYE	Greetings	Have a nice day

**Table 3:** Short and long slots proposed for the medication management scenario.

Short Slot Tags	Examples	Long Slot Tags	Examples
medication	ibuprofen	medication	ibuprofen
date	today, last Monday	date	today, last Monday
quantity	1, 2, one	duration	for 3 weeks
unitMedication	mL, cl, mg	posology	1 mg every 8 h
unitFrequency	days, hours, weeks		
timeExpressions	at breakfast		

**Table 4:** Results for slot filling with joint NLU (in %).

	Short Sentences		Long Sentences	
**Method**	**Short Slots**	**Long Slots**	**Short Slots**	**Long Slots**
SlotRefine	**76.4**	59.8	75.2	56.0
Stack-Prop.	**51.1**	33.1	49.5	31.6
SF-ID	**53.7**	29.3	49.9	31.0
Capsule-NLU	47.4	27.4	**53.1**	27.7
Slot-Gated	**57.8**	36.1	52.5	33.0
SLU-LM	38.7	19.5	**50.9**	20.2

**Table 5:** Results for intent detection with joint NLU (in %).

	Short Sentences		Long Sentences	
**Method**	**Short Slots**	**Long Slots**	**Short Slots**	**Long Slots**
SlotRefine	79.3	79.1	76.7	**81.4**
Stack-Prop.	59.2	**59.9**	**59.9**	59.7
SF-ID	**61.0**	60.5	56.8	57.6
Capsule-NLU	**45.0**	44.5	32.2	44.2
Slot-Gated	60.7	**61.0**	56.3	58.9
SLU-LM	46.9	45.6	**47.9**	46.6

**Table 6:** Results for sentence-level semantic frame with joint NLU (in %).

	Short Sentences		Long Sentences	
**Method**	**Short Slots**	**Long Slots**	**Short Slots**	**Long Slots**
SlotRefine	**68.6**	68.3	65.7	66.5
Stack-Prop.	36.9	**37.7**	35.9	36.9
SF-ID	**39.3**	35.9	32.2	34.3
Capsule-NLU	19.9	19.6	20.4	**21.5**
Slot-Gated	**40.6**	38.7	34.3	35.3
SLU-LM	**18.1**	15.7	17.3	14.1

**Table 7:** Errors in slot tagging for short slots (in %).

Slots	Sentences	SlotRefine	Stack-Prop.	SF-ID	Capsule-NLU	Slot-Gated	SLU-LM
Medication	Short	8.5	7.3	6.7	23.1	16.6	42.0
	Long	11.0	21.7	24.1	10.7	20.9	14.1
Date	Short	2.0	17.3	16.9	6.6	5.1	5.4
	Long	1.2	4.1	5.4	4.9	4.2	4.4
Quantity	Short	1.0	3.2	2.9	3.9	1.9	2.6
	Long	1.1	3.0	3.1	2.8	2.5	3.5
Unit	Short	0.8	1.9	1.5	1.9	1.0	1.8
Medication	Long	0.9	1.6	1.0	1.8	1.0	1.0
Unit	Short	1.0	2.4	1.6	0.9	1.1	3.0
Frequency	Long	0.6	1.6	1.7	2.1	1.2	2.8
Time	Short	0.4	1.1	0.4	0.3	0.5	1.0
Expressions	Long	0.1	0.5	0.4	0.5	0.5	1.0

**Table 8:** Errors in slot tagging for long slots (in %).

Slots	Sentences	SlotRefine	Stack-Prop.	SF-ID	Capsule-NLU	Slot-Gated	SLU-LM inTableHeader
Medication	Short	11.3	19.7	22.9	26.6	21.3	46.3
	Long	15.9	24.0	25.2	28.1	21.8	41.5
Date	Short	2.6	10.9	6.1	4.8	3.8	5.5
	Long	1.1	4.5	5.1	4.9	4.3	5.0
Duration	Short	1.6	2.7	4.6	4.3	4.5	10.0
	Long	2.2	3.4	3.0	4.3	4.4	6.4
Posology	Short	4.4	4.1	4.9	5.7	4.7	10.2
	Long	4.4	4.1	4.9	5.7	4.7	10.2

## Data Availability

The data are available in a publicly accessible repository. The datasets generated in this study are openly available in FigShare at 15 March 2022 https://doi.org/10.6084/m9.figshare.19361825.

## Abbreviations

The following abbreviations are used in this manuscript:

BLSTM	bi-directional long short-term memory
FN	false negatives
FP	false positives
IOB	inside, outside, beginning
LM	language modeling
NLU	natural language understanding
RNN	recurrent neural network
SLU	spoken language understanding
TP	true positives
